# Psychological stress of ICU nurses in the time of COVID-19

**DOI:** 10.1186/s13054-020-02926-2

**Published:** 2020-05-06

**Authors:** Xin Shen, Xiaoyue Zou, Xiaofeng Zhong, Jing Yan, Li Li

**Affiliations:** 1 Department of Critical Care Medicine, Zhejiang Hospital, 12 Lingyin Road, Hangzhou, 310013 China; 2 Emergency Care Unit, Huzhou First People’s Hospital, 158 Plaza Back Road, Huzhou, 313000 China; 3 Department of Critical Care Medicine, Wuhan Pulmonary Hospital, 28 Baofeng Road, Wuhan, 430000 China

As the coronavirus disease 2019 (COVID-19) pandemic accelerates, global health care systems have become overwhelmed [1], leading to great psychological pressure on nurses in the care of critically ill patients with COVID-19. Moreover, extreme incidents have occurred, such as suicide of nurses caring for critically ill patients, in Italy. In fact, the psychological problems are also common among nurses in Wuhan City, China. The Department of Critical Care Medicine, Wuhan Pulmonary Hospital, is the designated hospital for the treatment of severe patients with COVID-19. It has a total of 20 intensive care unit (ICU) beds and 102 nurses from the local hospital and other hospitals in the provinces and cities outside of Wuhan City. The critically ill patients receive mechanical ventilation. Most of them also need advanced life support, such as extracorporeal membrane oxygenation, continuous renal replacement therapy, and ventilation in the prone position. Front-line nurses experience huge workload, long-term fatigue, infection threat, and frustration with the death of patients whom they care. They also face anxiety or even misunderstanding among patients and their family members. In the early stage, nurses from other regions outside of Wuhan City did not communicate with each other and usually felt lonely. Additionally, they worried about their families and vice versa. All these factors have resulted in high psychological pressure among ICU nurses in Wuhan. We surveyed 85 ICU nurses in our ward and found that the main manifestations were decreased appetite or indigestion (59%), fatigue (55%), difficulty sleeping (45%), nervousness (28%), frequent crying (26%), and even suicidal thoughts (2%). Especially, young nurses with no experience of caring for critically ill patients face a greater psychological crisis. If these psychological problems are not solved effectively, they may not only lead to a decline in their immunity and increase the chances of COVID-19 infection but also have an adverse impact on the quality and safety of the medical care system [2–4]. Therefore, early measures were actively taken, and the following improvements were made (Table 1):

1. Each medical team included a psychologist, and early psychological assessments and interventions were conducted. Nurses were guided to develop a reasonable understanding of the pandemic and avoid excessive panic and anxiety. They were recommended to seek professional help from the psychologist if they felt psychological stress that was difficult to relieve.
2. Nurses were advised to get familiar with the working environment and working procedures as soon as possible. Working together with colleagues in a harmonious working environment can help relieve psychological stress.
3. They were asked to express emotions by talking, drawing, singing, and exercising. Some easy-to-learn methods, such as taking a deep breath, were encouraged to be used to relieve tension and anxiety. Relaxation exercise was recommended during work shifts under the guidance of mental health professionals.
4. They were advised to communicate with colleagues who had the same experience or similar feelings and then understand and heal each other. They were asked not to force herself or himself to forget unpleasant experiences. It was normal for them to not forget such experiences; they might even remember it for a lifetime.
5. Nurses were divided into 11 groups, and each group had a team leader to establish a WeChat online communication platform, enhance teamwork atmosphere and cohesion, and spread positive information, such as epidemic control and patient rehabilitation. Nurses were encouraged as more patients were effectively treated and the mortality rate declined.
6. Regular meetings were organized to find the sources of nurses’ psychological problems and targeted solutions. Most nurses from other provinces had no work experience in infectious diseases and were worried about getting infected at work. Education and training were strengthened accordingly, including the use of personal protective equipment, hand hygiene, ward disinfection, medical waste management, and occupational exposure management. If unsuccessful cure and poor prognosis of critically ill patients caused depression among nursing staffs, professional knowledge training was strengthened to deepen the understanding of the disease. More knowledge of the expert consensus for COVID-19 diagnosis and treatment was provided, and successful therapy cases were shared. If a nurse’s professional skills were not sufficient to take care of critically ill patients, he/she was allocated appropriate patients according to his/her actual nursing ability and provided special training as per the requirements. If it was a psychological problem caused by a physical overdraft at work, the shift working system was adjusted to ensure that nurses took rest as much as possible, such as shift rotation reduced from 6 to 4 h.
7. A professional consultation team was set up, mainly including mental health professionals. Regular remote mental health training and guidance, individualized psychotherapy, or appropriate medical intervention was provided to nurses through lectures, group counseling, individual counseling, online platforms, and psychological hotlines.
8. The social support system was improved. The original working unit established a care and support group to strengthen humanitarian care. Their leaders, colleagues, and volunteers regularly visited the family members of the nursing staffs to find and resolve their worries in a timely manner. Regular chat and exchange with family and former colleagues were organized through WeChat videos.

**Table 1:** Psychological stress of ICU nurses in the time of COVID-19

	Problems	Solutions
1	Anxiety regarding unfamiliar working environment and processes.	Establish a communication mechanism with local medical staff to get familiar with the working environment and working procedures as soon as possible.
2	Lack of work experience in infectious diseases.	Allocate appropriate patients according to the actual nursing ability and provide necessary special training.
3	Worry about getting infected.	Enhance education and training, including personal protective, hand hygiene, ward disinfection, medical waste management, and occupational exposure management.
4	Huge workload and long-term fatigue.	Adjust work shift to ensure nurses to have plenty of rest.
5	Depression due to unsuccessful cure of critically ill patients.	1) Equip each medical team with a psychologist for early psychological assessments and interventions. 2) Strengthen professional training to deepen the understanding of the disease. 3) Share successful therapy cases. 4) Actively express emotions to relieve tension and anxiety. 5) Perform relaxation exercise under the guidance of the mental health professionals.
6	Worry about their families, and vice versa.	1) Communicate with colleagues who have the same experience or similar feelings. 2) Regularly chat and exchange with family through WeChat videos. 3) Establish a social care and support group to find and resolve worries accordingly. 4) Set up a professional team to provide remote mental health training and guidance, individualized psychotherapy, or appropriate medical intervention to nurses in multiple ways.

In summary, through the early assessment and active resolution of psychological stress, nurses experienced no adverse events during the fight against COVID-19. Of course, the long-term psychological changes in nurses needed a regular follow-up. Hence, it is recommended to address the psychological problems of ICU nurses who care for patients with COVID-19 and take action as soon as possible to relieve the psychological pressure on these nurses. Our experience may serve as a valuable reference while designing psychological health interventions for nurses in future large-scale public health emergencies.

## Data Availability

Not applicable.
